# Sexually Transmitted Disease–Related Reddit Posts During the COVID-19 Pandemic: Latent Dirichlet Allocation Analysis

**DOI:** 10.2196/37258

**Published:** 2022-10-31

**Authors:** Amy K Johnson, Runa Bhaumik, Debarghya Nandi, Abhishikta Roy, Supriya D Mehta

**Affiliations:** 1 The Potocsnak Family Division of Adolescent and Young Adult Medicine Ann & Robert H. Lurie Children's Hospital of Chicago Chicago, IL United States; 2 Feinberg School of Medicine Northwestern University Chicago, IL United States; 3 School of Public Health University of Illinois at Chicago Chicago, IL United States; 4 Rush University Chicago, IL United States

**Keywords:** infodemiology, Latent Dirichlet Allocation, natural language processing, Reddit, sexually transmitted infections, surveillance, social media, COVID-19, social media content, content analysis, health outcome, infoveillance, health information, sexually transmitted disease, STD

## Abstract

**Background:**

Sexually transmitted diseases (STDs) are common and costly, impacting approximately 1 in 5 people annually. Reddit, the sixth most used internet site in the world, is a user-generated social media discussion platform that may be useful in monitoring discussion about STD symptoms and exposure.

**Objective:**

This study sought to define and identify patterns and insights into STD-related discussions on Reddit over the course of the COVID-19 pandemic.

**Methods:**

We extracted posts from Reddit from March 2019 through July 2021. We used a topic modeling method, Latent Dirichlet Allocation, to identify the most common topics discussed in the Reddit posts. We then used word clouds, qualitative topic labeling, and spline regression to characterize the content and distribution of the topics observed.

**Results:**

Our extraction resulted in 24,311 total posts. Latent Dirichlet Allocation topic modeling showed that with 8 topics for each time period, we achieved high coherence values (pre–COVID-19=0.41, prevaccination=0.42, and postvaccination=0.44). Although most topic categories remained the same over time, the relative proportion of topics changed and new topics emerged. Spline regression revealed that some key terms had variability in the percentage of posts that coincided with pre–COVID-19 and post–COVID-19 periods, whereas others were uniform across the study periods.

**Conclusions:**

Our study’s use of Reddit is a novel way to gain insights into STD symptoms experienced, potential exposures, testing decisions, common questions, and behavior patterns (eg, during lockdown periods). For example, reduction in STD screening may result in observed negative health outcomes due to missed cases, which also impacts onward transmission. As Reddit use is anonymous, users may discuss sensitive topics with greater detail and more freely than in clinical encounters. Data from anonymous Reddit posts may be leveraged to enhance the understanding of the distribution of disease and need for targeted outreach or screening programs. This study provides evidence in favor of establishing Reddit as having feasibility and utility to enhance the understanding of sexual behaviors, STD experiences, and needed health engagement with the public.

## Introduction

More than 2.5 million cases of chlamydia, gonorrhea, and syphilis were reported in 2019, with sexually transmitted diseases (STD) cases reaching an all-time high for the sixth consecutive year in the United States [1]. STDs are common and costly, impacting approximately 1 in 5 people annually and accounting for US $16 billion in annual health care costs [2]. New data from the Centers for Disease Control and Prevention demonstrate that during the start of the COVID-19 pandemic (from March to April 2020), reported STD cases dramatically decreased compared to the same time in 2019. At that point, the current cumulative totals for STD cases compared to 2019 were 1% lower for primary and secondary syphilis, 7% lower for gonorrhea, and 14% lower for chlamydia [3]. Although case reports were lower for the first part of 2020, cases rebounded later in the year and were on track to surpass 2019 totals [3].

Multiple factors likely contributed to the observed decrease in reported STD cases during the early phases of the COVID-19 pandemic. Restrictions of in-person clinic visits resulted in reduced screening of asymptomatic patients. The Centers for Disease Control and Prevention provided guidance for sexual health services to prioritize patients based on symptoms and risk, along with delaying routine screening until after the emergency response [4]. Many health department staff were redeployed from STD tracking to COVID-19 contact tracing and control [5]; 57% of disease intervention specialists reported that they were reassigned from STD to COVID-19 services, limiting the workforce available to provide STD prevention, screening, and treatment [5]. Finally, national stay-at-home orders were issued during phases of the pandemic that were designed to reduce the spread of COVID-19 but may also have reduced STD transmission by reducing sexual behavior outside of the household, limiting the number of new sexual partners, and restricting sexual networks [6].

Recent estimates indicate that 80% of all internet users report accessing health information on the web [7]. As the internet can be accessed anonymously and at any time, users can seek STD information and resources confidentially, which may facilitate more frequent and open disclosure of symptoms and exposure experiences [8]. Reddit, the sixth most used internet site in the world, is a user-generated social media discussion platform that may be useful in monitoring discussion about STD symptoms and exposure [9]. Reddit is considered one of the most authentic web spaces as there are safeguards against “bot accounts” and rich communication occurs without the barrier of requiring demographic or identifying information to join [10,11]. Prior health research has established that Reddit is an acceptable platform to conduct scientific investigations [10,12,13]. Topic-specific Reddit discussions (subreddits) dedicated to discussing sexual health and STDs may provide valuable insight to exposure, symptoms, testing, and sexual behavior during the COVID-19 pandemic. Prior analyses of Reddit discussion content have been conducted across different diseases and health conditions, including smoking cessation, atopic dermatitis, suicide, and pregnancy [10-13]. To derive meaningful and replicable information from Reddit discussion content, the complexity of high-volume text data needs numerical structure implemented in an unbiased way. Latent Dirichlet Allocation (LDA) is a natural language processing method that identifies common words and topics in text and allows experts to assess common themes among findings [14]. This study sought to define and identify patterns and insights into STD-related discussions on Reddit via LDA over the course of the COVID-19 pandemic. Our team hypothesized that there would be an increase in the volume of STD-related posts on Reddit and the variation of topics during the COVID-19 pandemic compared to the prepandemic period due to behavior changes during the COVID-19 pandemic.

## Methods

### Ethics Approval

The study protocol was determined to be nonhuman subjects research by the Ann & Robert H. Lurie Children’s Hospital Institutional Review Board (IRB#2022-4964) because of the use of publicly available, nonidentifiable data.

### Data Extraction

**Figure 1:**
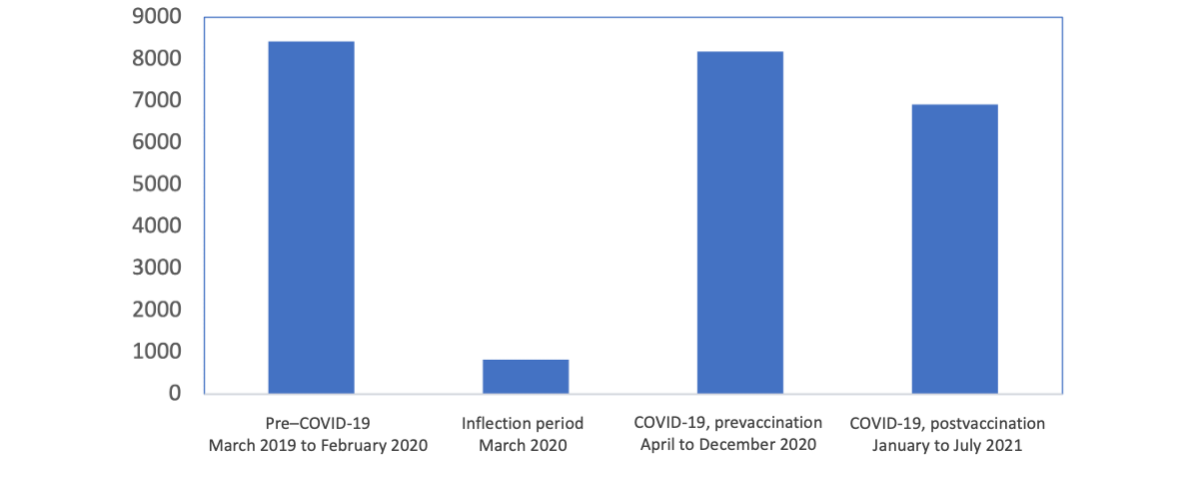
Distribution of volume of posts for study time periods from March 2019 to July 2021, resulting in a total of 24,311 posts.

This study used publicly available data from the web-based discussion forum, Reddit. Reddit is an anonymous social media site that is user-generated and discussion-based. The site is organized into “subreddits” that are content-specific. Posts were extracted from 2 subreddits: “STD” (r/STD) and “sexual health”(r/sexualhealth). However, due to the small number of posts in r/sexual health, we only used the subreddit r/STD in our analysis. The *pushshift* Reddit application programming interface was used for searching Reddit comments and submissions [15]. Reddit’s official application programming interface (Reddit 2021) was used to collect posts and associated metadata (date) from r/STD and r/sexualhealth from March 2019 to July 2021, resulting in 24,311 posts [10]. Only English-language posts were included in the analysis. Figure 1 displays the number of posts that were extracted from each subreddit for the time frames used in the analysis. “Pre–COVID-19” was defined as ranging from March 2019 to February 2020 (8421 posts); “COVID-19, prevaccination” was defined as ranging from April to December 2020 (8169 posts); “COVID-19, postvaccination” was defined as ranging from January to July 2021 (6908 posts); and an inflection period was defined as March 2020 (813 posts). Based on the most current literature, we reasonably supposed the absence of seasonality in sexually transmitted infection (STI) cases [16,17].

### Data Preprocessing

Data preprocessing steps were conducted following common approaches in natural language processing [18]. Preprocessing eliminates some of the inconsistencies in the data and reduces the content to useable text. In all, 4 preprocessing steps were completed on each line from the text file to extract and clean each title, body, and comment separately: (1) the removal of URLs, (2) tokenization, (3) punctuation and stop word removal, and (4) lemmatization [19-21].

### Statistical Analysis

#### LDA Topic Modeling

**Figure 2:**
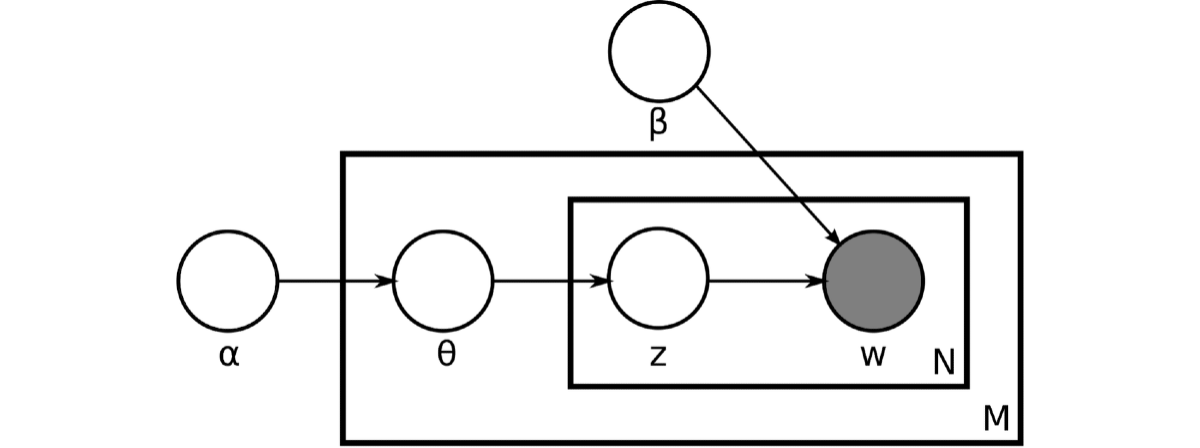
Latent Dirichlet Allocation in plate notation (adapted from Blei et al [14]).

We used an increasingly popular topic modeling method, LDA, to conduct a text analysis identifying the most common topics discussed in the Reddit posts [22]. LDA is a statistical generative model that discovers latent semantic topics in large collections of text documents (posts in our study), where each document results from random mixtures over latent topics and each topic is characterized by a distribution over words. The model is presented in plate notation in Figure 2 [14]. Both the topics and words have a Dirichlet prior distribution, respectively, with α being the parameter of the per-document Dirichlet prior on the topics, and β being the parameter of the per-word Dirichlet prior on the words.  θ_m_ is the topic distribution for document m. ϕ_k_ is the word distribution for topic k. Z_nm_ is the topic for the nth word in the mth document. W_nm_ is the actual nth word in the mth document. Considering the nature of its structure, LDA is a multiple-level hierarchical Bayesian model.

To conduct the LDA, we converted the corpus to a document-term matrix, comprising rows representing original posts and columns representing each word in the corpus. Each cell in the document-term matrix contains the frequency of times a specific word (defined by the column) occurred in a specific post (defined by the row). From this document-term matrix, the entire corpus was represented, including patterns of words that commonly occur together within the same post. We used the *gensim* library to perform LDA model estimation, which determined sets of words that appeared frequently together in posts across sexual health subreddits [19].

**Figure 3:**
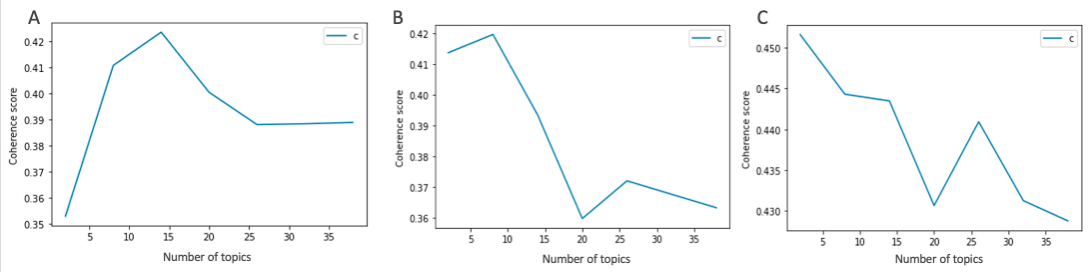
Latent Dirichlet Allocation topic modeling on multiple time periods: (A) pre–COVID-19, (B) prevaccination, and (C) postvaccination.

The LDA model then outputs a topic-document matrix, representing the relative importance of each topic in each document. Models were applied to pre–COVID-19 posts from March 2019 to February 2020 (8421 posts), prevaccination posts from April to December 2020 (8169 posts), and postvaccination posts from January to July 2021 (6908 posts; Figure 3). For topic modeling, we excluded posts for the inflection period (March 2020; 813 posts).

A key process in LDA is to estimate the optimal number of topics. To estimate the number of topics, we used the topic coherence index, which is the most consistent measure of human interpretability [23]. Topic coherence measures score a single topic by measuring the degree of semantic similarity between high-scoring words in the topic. These measurements help distinguish between topics that are semantically interpretable topics and topics that are artifacts of statistical inference. The higher the topic coherence score, the better the quality of the model. To avoid overfit and sparsity and improve inference, we selected the number of topics as 8. Topics were reviewed and labeled independently by 2 experts in STD epidemiology and control (AKJ and SDM). Once independent review was completed, labels were discussed until consensus was reached, resulting in 100% agreement.

#### Word Cloud

A word cloud is a text visualization technique that focuses on the frequency of words and correlates the size and opacity of a word to its frequency within a text body. The output is usually an image that depicts different words in different sizes and opacities relative to the word frequency. Separate frames were created for posts containing the following terms: chlamydia, gonorrhea, syphilis, gonorrhea/discharge/dysuria, and syphilis/chancre/ulcer. After data preprocessing was completed, each string was passed to the *WordCloud* function in Python to generate a word cloud [24]. For *WordCloud* visualization, we chose 3 etiologic terms (chlamydia, gonorrhea, and syphilis) and 3 of the most common terminologies from the topic search: herpes/herpes simplex virus (HSV)/human papillomavirus (HPV; as a single topic, due to correlations), diagnosis/testing, and STI/STD. Each separate word cloud was formed by searching each word in the topic.

#### Spline Regression Plots

Spline regression modeling has become popular in applied clinical research. Modern biostatistics makes use of spline regression to model smooth functions such as time series, cumulative effects, and frequency distributions and in survival analysis. Spline regression is used to overcome the difficulties of linear and polynomial regression algorithms. In linear regression, the entire data set is considered once. Polynomial regression can express a particular amount of curvature in a nonlinear relationship, but in spline regression (a nonparametric regression), the data set is divided into bins. Each bin of the data is fitted with separate models. The points where the data are divided into bins are called knots. In simpler words, splines are piecewise polynomial functions. To identify patterns in the change of the proportion of posts related to certain search terms relative to the total number of posts in a particular month over the entire study period (spanning from March 2019 to July 2021), a spline plot was created. The pre-COVID-19; inflection; COVID-19, prevaccination; and COVID-19, postvaccination periods were highlighted on the plots for a better understanding of search trends across time. The plots were created using *ggplot2* package in R statistical software (R Foundation for Statistical Computing) [25]. For spline regression, we used a cubic B-spline basis with 2 boundary knots and 1 interior knot placed at the median of the observed data values. As with the word clouds, we created 3 plots based on etiology (chlamydia, gonorrhea, and syphilis) and 3 plots based on common topics (diagnose/test/tested, herpes/HSV/HPV, and gonorrhea/dysuria/discharge). A detailed review of spline regression using R software can be found in Perperoglou et al [26].

## Results

### Reddit Posts

**Figure 4:**
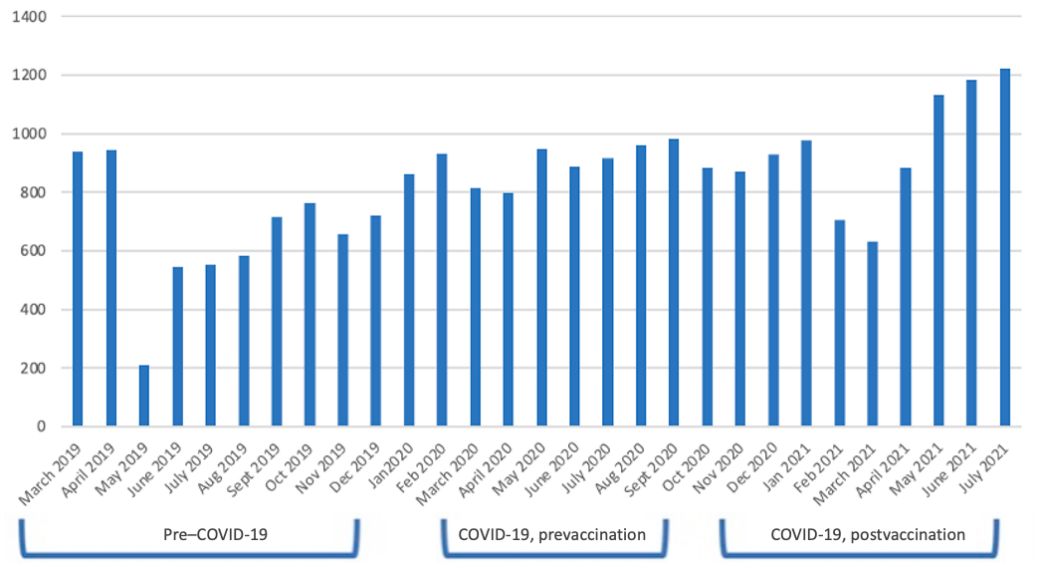
Average number of Reddit posts per month, by period.

Of the 24,311 posts, the average number of posts per month were 701.75 during the pre–COVID-19 period; 907.67 during the COVID-19, prevaccination period; and 863.50 during the COVID-19, postvaccination period, but there was substantial variability from month to month and within each time period. The average number of posts per month per period demonstrated growth in subreddit volume during COVID-19. Figure 4 displays the number of posts per month by observation period. May 2019 consisted of 210 posts and August 2021 consisted of 169 posts, which were 2 of the lowest volumes recorded and were both preceded by 2 months of high-volume posts.

**Figure 5:**
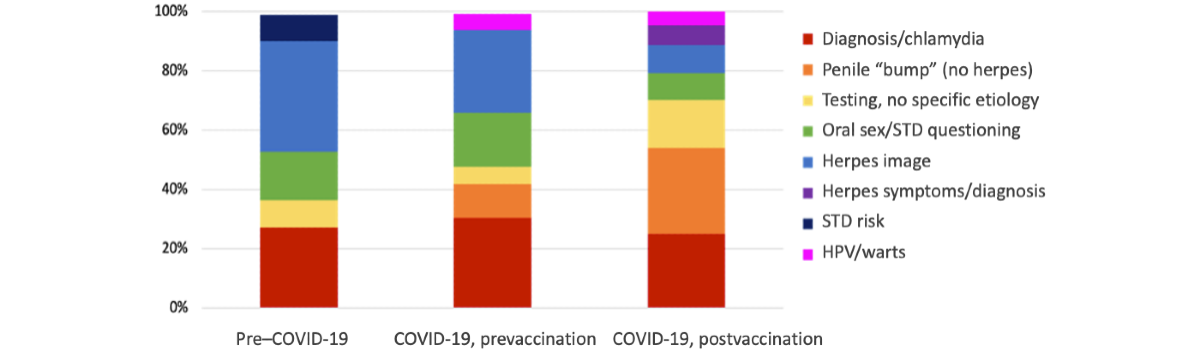
Distribution of posts: the proportion of documents that are assigned to each topic. STD: sexually transmitted disease; HPV: human papillomavirus.

LDA topic modeling showed that with 8 topics for each time period, we achieved high coherence values (pre–COVID-19=0.41, prevaccination=0.42, and postvaccination=0.44). Figure 5 shows the distribution of topic posts in pre–COVID-19, prevaccination, and postvaccination “STD” and “sexual health” subreddits over the 8 topics extracted using LDA. Although most topic categories remained the same over time, the relative proportion of topics changed and new topics emerged. In the pre–COVID-19 period, a general category of “STD Risk” emerged with no specific etiology or mention of symptoms with words such as “negative” and “exposure” in the top 10 terms associated with the topic (Table 1). “HPV” and “warts” as terms did not appear in the pre–COVID-19 period. There was specific language surrounding herpes symptoms (eg, “outbreak”) and diagnosis (eg, testing and positive or negative) and the introduction of “HSV” in the postvaccination period, whereas words used in conjunction with herpes in previous periods were primarily related to images and nonspecific symptoms (eg, “redness” and “bumps”; Table 2). Moreover, although the “herpes image” topic category included nonspecific symptoms (eg, “bump” and “redness”), this categorization diverged during COVID-19 periods, with a topic category emerging for penile “bump” without the mention of herpes. In the postvaccination period, the “oral sex/STD questioning” topic included the term “penis”; although this topic existed in the other 2 periods, it did not include “penis” as a top 10 term (Table 3).

**Table 1:** Pre–COVID-19 topics and the top 10 terms derived from a Latent Dirichlet Allocation model created from 3 different time periods: pre–COVID-19, prevaccination, and postvaccination.

Topic	Top 10 terms
Testing, no specific etiology	day, test, week, negative, feel, take, pain, still, exposure, also
Herpes image	com, imgur, penis, sex, STD^a^, help, really, day, condom, herpes
Oral sex/STD questioning	sex, oral, day, ago, STD, know, condom, unprotected, time, penis
Penile “bump”	com, imgur, bump, look, red, penis, herpes, week, day, spot
Diagnosis/doctor (results)	test, say, back, doctor, look, hepatitis, come, herpes, throat, negative
Diagnosis/chlamydia	test, sex, chlamydia, week, month, symptom, come, back, time, partner

^a^STD: sexually transmitted disease.

**Table 2:** Prevaccination topics and the top 10 terms derived from a Latent Dirichlet Allocation model created from 3 different time periods: pre–COVID-19, prevaccination, and postvaccination.

Topic	Top 10 terms
Oral sex/STD^a^ questioning	sex, oral, come, week, test, day, know, back, STD, time
HPV^b^/warts treatment, herpes questioning	test, sex, HPV, time, year, condom, month, ago, last, say
Diagnosis/chlamydia	test, take, symptom, day, week, know, say, back, chlamydia, doctor
Testing, no specific etiology image	day, month, feel, start, sex, doctor, pain, take, test, thing
Diagnosis (results)	test, positive, negative, sex, chlamydia, result, herpes, partner, day, month
Penile “bump” symptom (no pictures, no herpes)	com, imgur, bump, help, penis, look, know, pimple, hurt, think
Herpes image	imgur, com, herpes, bump, red, penis, help, day, look, month

^a^STD: sexually transmitted disease.

^b^HPV: human papillomavirus.

**Table 3:** Postvaccination topics and the top 10 terms derived from a Latent Dirichlet Allocation model created from 3 different time periods: pre–COVID-19, prevaccination, and postvaccination.

Topic	Top 10 terms
Herpes symptoms/diagnosis	test, herpes, HSV^a^, sex, know, outbreak, negative, positive, genital, risk
Herpes image	com, imgur, herpes, sex, look, help, remove, know, oral, bump
HPV^b^/warts treatment, herpes questioning	wart, herpes, ibb_co, com, www_reddit, comment, remove, HPV, month, skin
Diagnosis/chlamydia	day, month, feel, start, sex, doctor, pain, take, test, thing
Testing, no specific etiology	test, remove, STD, day, sex, week, negative, help, time, oral
Penile “bump” symptom (no pictures, no herpes)	penis, bump, sex, day, know, STD^c^, feel, look, condom, time
Oral sex/STD questioning	sex, know, test, week, say, think, time, symptom, oral, tell
Penile “bump” symptom	bump, com, look, imgur, week, ago, day, penis, red, notice

^a^HSV: herpes simplex virus.

^b^HPV: human papillomavirus.

^c^STD: sexually transmitted disease.

### Word Clouds

**Figure 6:**
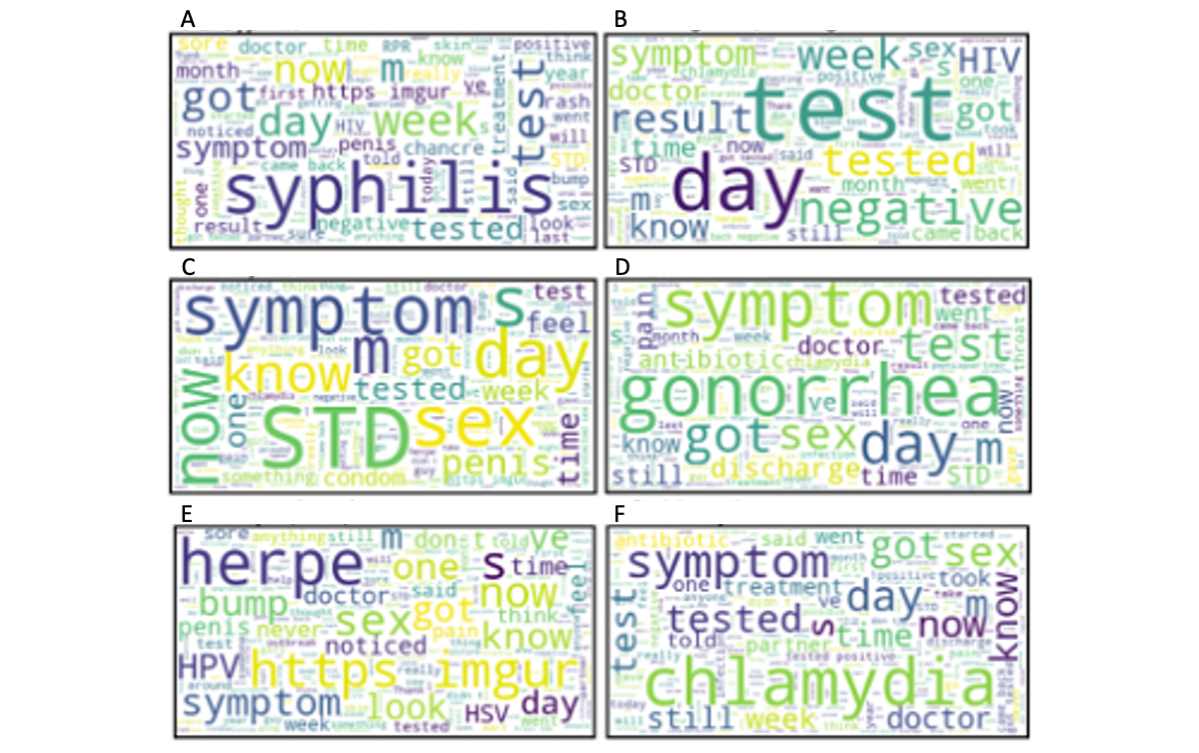
Word clouds by key term: (A) syphilis, (B) diagnosis/testing, (C) STI/STD, (D) gonorrhea, (E) herpes/HSV/HPV, and (F) chlamydia. HPV: human papillomavirus; HSV: herpes simplex virus; STD: sexually transmitted disease; STI: sexually transmitted infection.

Although the terms in the topic models listed above are informative, we used *WordCloud* visualizations to better understand the relative importance of these words within each topic based on etiology and general terms over the study period. The terms that appear larger appeared more frequently within the topic, whereas the terms in smaller font appeared less frequently. Figure 6A-F displays the word clouds for 6 specific topics; for example, Figure 6E displays terms clustered with herpes/HSV/HPV such as “imgur” (denoting that a picture was uploaded), “bump,” “pain,” and “outbreak.”

### Spline Regressions

**Figure 7:**
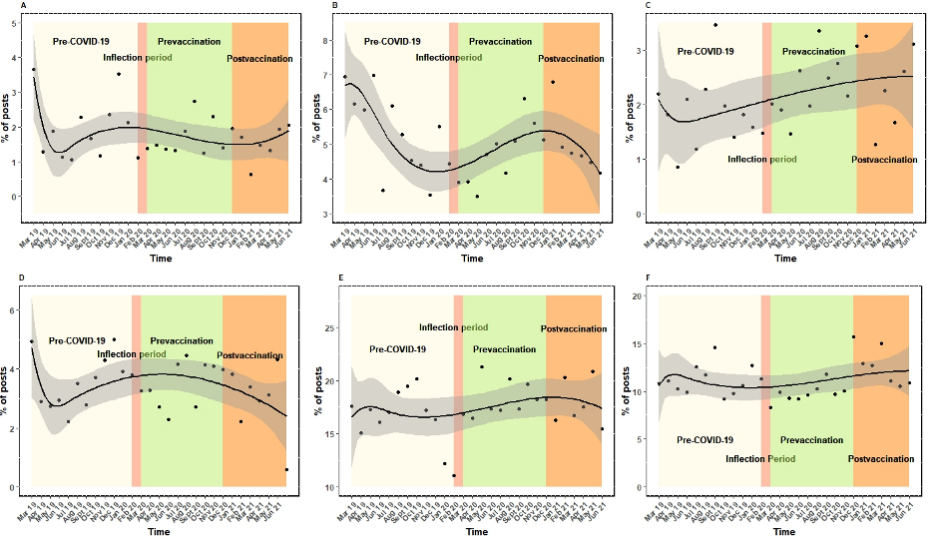
Percentage of Reddit posts containing specific key terms from March 2019 to July 2021: (A) gonorrhea, (B) chlamydia, (C) syphilis, (D) gonorrhea/dysuria/discharge, (E) herpes/HSV/HPV, and (F) diagnose/test/tested. HPV: human papillomavirus; HSV: herpes simplex virus.

As shown in the series of spline regressions in Figure 7A-F, there is some variability in the percentage of posts by key terms over the study periods. Although some regressions are “flat” (ie, uniform) across the study periods, others display variability that coincides with the COVID-19 periods. For example, Figure 7F displays the regression for posts with the key terms “diagnose/test/tested.” There is some variability in the percentage of posts in the different COVID-19 periods, with the pre–COVID-19 and postvaccination volumes being similar to each other and the prevaccination period having a lower percentage of posts. We tested the significance of the differences between the pre–COVID-19 and post–COVID-19 frequency of posts for 6 key terms (Table 4). There are statistically significant differences for herpes and syphilis, with post–COVID-19 frequencies being higher than pre–COVID-19 frequencies.

**Table 4:** Comparing the differences between the pre–COVID-19 and post-COVID-19 frequencies of posts.

Key term	Pre–COVID-19, frequency	Post–COVID-19, frequency	*P* value
Chlamydia	35.27	43.47	.08
Gonorrhea	14.25	15.56	.64
Herpes	118.75	160.47	.01
Syphilis	12.92	21.23	.005
Test/diagnosis	78	97.65	.08
Gonorrhea/dysuria	25.33	30.47	.28

## Discussion

### Principal Findings

Our study provides evidence that there was an increase in the volume of STD-related posts during the COVID-19 pandemic periods and there were changes in the topics posted in STD-related subreddits from pre–COVID-19 through COVID-19, prevaccination and COVID-19, postvaccination periods. The changes in topics discussed likely relate to behavior changes due to COVID-19–related lockdowns, restrictions on in-person gatherings, and the closing of nonessential medical services [27]. Regardless of lockdown status, people still engage in sexual behavior (eg, condomless sex) that will expose them to STDs. However, with the reduction of STD testing or treatment, these cases are not reflected in surveillance numbers. It is important to understand the sexual health experiences of communities, including symptoms, questions, and behavior patterns, to plan for screening and treatment options.

Our results found that “STD risk” as a topic and general “risk” terms as words only appeared in the pre–COVID-19 time period, whereas “HPV” and “warts” only appeared in the COVID-19, prevaccination and postvaccination periods. During the pre–COVID-19 time period, users generated posts related to general STD risk and sexual behavior, seeking advice and support for understanding STD exposure risk for specific sexual behavior or partnership choices. During the 2 COVID-19 periods, this general “STD risk” topic no longer appeared, demonstrating a difference in content—moving from general discussions to specific symptom or etiology-based posts. During the 2 COVID-19 periods, HPV/warts emerged as a topic. This finding may be due to increased effort to self-diagnosis symptoms experienced as a result of limited access to diagnostic services. Although reported STD cases declined during the initial lockdown period, cases reported in 2020 quickly rebounded and exceed the case numbers in 2019 [3].

Our study’s use of Reddit is a novel way to gain insights into STD symptoms experienced, potential exposures, testing decisions, common questions, and behavior patterns (eg, during lockdown periods). For example, reduction in STD screening may result in observed negative health outcomes due to missed cases, which also impacts onward transmission. The reduction in access to STD testing and treatment during COVID-19 intensified existing barriers to sexual health care, including stigma, judgement, cost, and accessibility [28]. It is important that STD services be maintained, either through telehealth and in-home testing options or via clinic services with COVID-19 mitigation procedures in place (screening, masking, and social distancing).

As Reddit use is anonymous, users may discuss sensitive topics with greater detail and more freely than in clinical encounters. The sexual health subreddits had an average volume of unique posts ranging from approximately 700 to 900 per month; thus, Reddit is a frequently used source of information that could guide the understanding of the behavior, symptoms, and common questions of patients. Sexual health care workers should consider collaboration with Reddit or other social media outlets to leverage the potential benefits of these platforms (anonymous, free, and rapid response) while mitigating harm (incorrect diagnoses and faulty recommendations) [29].

### Limitations

Study results should be interpreted while considering the following limitations. LDA is an unsupervised approach with no gold standard to compare to. However, we analyzed the LDA output qualitatively with the use of 2 independent coders and reached 100% consensus on manual topic labels. As we used posts from an open web-based forum, we were unable to validate users; however, there is little incentive to be dishonest or to post false information on health-related subreddits. Reddit users tend to be younger and are more likely to be male compared to the larger US population; however, other demographic trends (eg, race/ethnicity) mirror the distribution in the United States [30]. As men and Black or African American and Latino communities are often underrepresented in STI case data, it is important to gain an understanding of their sexual health needs and experiences via alternative data sources [30]. Finally, the precise location of Reddit users are unknown. Although we were able to extract posts limited to the United States and those in the English language, we cannot pinpoint post volume by specific state or local jurisdiction.

### Conclusion

This study demonstrates Reddit as having feasibility and utility to enhance the understanding of sexual behaviors, STD experiences, and needed health engagement with the public. It is important to prioritize efforts to reduce the spread and impact of STDs through surveillance, screening, and treatment. The COVID-19 pandemic and subsequent stay-at-home orders highlight a critical need for increased access to STD clinics and STD information. Data from anonymous Reddit posts may be leveraged to enhance the understanding of the distribution of disease and need for targeted outreach or screening programs.

## Abbreviations

HPV: human papillomavirus
HSV: herpes simplex virus
LDA: Latent Dirichlet Allocation
STD: sexually transmitted disease
STI: sexually transmitted infection
